# Altered locus coeruleus functional connectivity in migraine without aura based on resting-state functional magnetic resonance imaging

**DOI:** 10.22514/jofph.2026.054

**Published:** 2026-07-12

**Authors:** Wanting Han, Yaxuan Wang, Xiaobin Huang, Fengfang Li, Di Zhang, Yujia Gao, Lindong Liu, Tong Fu, Xinying Wu

**Affiliations:** ^1^Department of Radiology, Nanjing First Hospital, Nanjing Medical University, 210006 Nanjing, Jiangsu, China

**Keywords:** Migraine without aura, Resting-state functional magnetic resonance imaging, Functional connectivity, Locus coeruleus

## Abstract

**Background**: Accumulating evidence indicates that the locus coeruleus (LC) plays a pivotal role in pain modulation and related network dysfunction in migraine. This study aimed to examine functional connectivity (FC) between the LC and other brain regions in patients with migraine without aura (MwoA) compared with healthy controls (HCs). **Methods**: A total of 55 patients with MwoA and 50 age-, sex-, and education-matched HCs underwent resting-state functional magnetic resonance imaging (fMRI). Seed-to-voxel whole-brain FC analysis was performed using the bilateral LC as seed regions. Different clinical and neuropsychological assessments are included. Pearson correlation analysis was used to determine the relationships between altered FC and clinical variables, and receiver operating characteristic (ROC) analysis was further performed to evaluate the discriminatory performance of significant FC measures. **Results**: Compared with HCs, patients with MwoA exhibited increased FC between the left LC and the right superior temporal gyrus (STG)/left cerebellar posterior lobe (CPL), as well as between the right LC and the left STG/left inferior occipital gyrus (IOG) (*p* < 0.05). Decreased FC was observed between the bilateral LC and the right superior frontal gyrus (SFG) (*p* < 0.05). Positive correlations were identified between disease duration and FC of the left LC–right STG connection (*p* = 0.012, *r* = 0.341), and between SDS scores and FC of the right LC–left IOG connection (*p* = 0.011, *r* = 0.344). ROC analysis demonstrated that FC between the right LC and right SFG had the best discriminatory performance (sensitivity 81.82%, specificity 68.00%). **Conclusions**: Patients with MwoA exhibited altered LC-related resting-state FC involving brain regions associated with pain processing, sensory integration, and emotional regulation. By separately characterizing the whole-brain connectivity patterns of the bilateral LC, this study extends previous LC-related imaging findings in migraine and provides a more refined description of LC-centered network dysfunction in MwoA.

## 1. Introduction

Migraine is a complex and highly disabling neurological disorder characterized by recurrent attacks of moderate to severe headache, often accompanied by nausea, vomiting, photophobia, and phonophobia [1]. According to the third edition of the International Classification of Headache Disorders (ICHD-3) [2], migraine without aura (MwoA) represents the most prevalent subtype of migraine. Although accumulating neuroimaging evidence has shown that migraine is associated with alterations in brain network organization, the neural mechanisms underlying MwoA remain incompletely understood [3, 4]. Resting-state functional connectivity (FC), which reflects temporal correlations between spatially distinct brain regions, provides a useful framework for investigating these network-level abnormalities [5].

Among the brainstem nuclei implicated in migraine, the locus coeruleus (LC) has received increasing attention because of its central role in pain modulation, arousal, stress regulation, and autonomic function [6, 7]. As the principal noradrenergic nucleus of the brain, the LC sends widespread projections to cortical, subcortical, and cerebellar regions, placing it in a key position to influence nociceptive processing and multisensory integration. Consistent with this view, previous neuroimaging studies have reported LC-related connectivity abnormalities in migraine. Altered hypothalamic FC with autonomic circuits and the LC has been demonstrated in migraine, suggesting the involvement of LC-related brainstem-diencephalic networks in this disorder [8]. More recently, increased LC functional connectivity has been reported in patients with MwoA [9], while altered LC functional network characteristics have also been observed in patients with comorbid migraine and insomnia [10]. Structural neuroimaging evidence has further indicated abnormalities in the LC and related brainstem regions in patients with migraine [11]. Taken together, these findings support a potential role of LC-centered network dysfunction in migraine.

In addition to its role in pain processing, the LC is closely linked to networks involved in emotional regulation and cognitive function. Many patients with migraine experience comorbid symptoms, including anxiety, depression, and memory deficits, which may be related to dysregulated LC connectivity [12]. These emotional and cognitive disturbances further complicate the clinical profile of MwoA and highlight the need for a more comprehensive understanding of its neurobiological basis.

However, several issues remain unresolved. Existing LC-related imaging studies in migraine remain limited, and some have focused on specific clinical subgroups rather than MwoA itself. Moreover, the whole-brain FC profiles of the bilateral LC in patients with MwoA have not yet been fully characterized, while the clinical significance of altered LC-related FC in MwoA, particularly its associations with disease burden and affective symptoms, remains insufficiently understood. Further investigation is therefore warranted to clarify LC-centered functional network alterations in MwoA.

In the present study, we examined whole-brain resting-state FC of the bilateral LC in patients with MwoA compared with HCs. We further evaluated the associations between altered LC-related connectivity and clinical variables and performed an exploratory receiver operating characteristic (ROC) analysis to assess the preliminary discriminatory relevance of significant FC measures. We hypothesized that patients with MwoA would exhibit altered LC connectivity with regions involved in pain modulation, sensory integration, and emotional regulation, and that some of these alterations would be associated with clinical characteristics of the disorder. The novelty of the present study does not lie in claiming the first evidence of LC involvement in migraine, but rather in providing a more refined characterization of bilateral LC-centered whole-brain FC abnormalities in a well-defined MwoA cohort by separately examining the left and right LC, while further exploring their clinical associations and preliminary classification relevance.

## 2. Methods

### 2.1 Participants

A cross-sectional analysis was conducted between May 2018 and June 2023 and included 55 individuals diagnosed with MwoA and 50 HCs. This study was approved by the Human Research Ethics Committee of Nanjing First Hospital (No. KY20200301-16), and written informed consent was obtained from all participants before MRI scanning. Participants were recruited from the Department of Neurology and the Department of Pain Outpatient Clinic of Nanjing First Hospital. The inclusion criteria for patients with MwoA were based on the International Classification of Headache Disorders, Third Edition, beta version (ICHD-3 beta) [2]. Exclusion criteria included a history of neurological or psychiatric disorders, serious systemic diseases such as cancer, excessive use of psychoactive medications, and any contraindications to MRI. To minimize the potential effects of pain and medication on blood oxygen level-dependent (BOLD) signal fluctuations, all patients were required to be free of headache attacks and medication use for at least 72 hours before scanning. In addition, patients were followed up 3 days after scanning to confirm that they had remained migraine-free throughout this period. Moreover, to reduce the potential effects of hormonal fluctuations on cortical excitability, female participants were scanned during the mid-cycle phase, and women who were pregnant or breastfeeding were excluded.

Age-, sex-, and education-matched HCs were recruited from the local population. These individuals had no personal or family history of migraine or other headache disorders and either experienced no headaches or had only infrequent tension-type headaches, defined as fewer than one episode per month. Participants who had received any treatments that could potentially affect the central nervous system were excluded from the study.

All participants were right-handed according to self-report. Clinical and neuropsychological assessments were completed before MRI scanning. All participants completed the Self-Rating Anxiety Scale (SAS), Self-Rating Depression Scale (SDS), and Montreal Cognitive Assessment (MoCA). In addition, patients with MwoA completed the Headache Impact Test-6 (HIT-6) and Migraine Disability Assessment (MIDAS). Anxiety and depressive symptoms were assessed using the SAS and SDS, respectively. Both instruments are self-report scales developed by Zung [13, 14], and they were completed by the participants themselves under the guidance of trained researchers to ensure the completeness and accuracy of the responses. Higher SAS and SDS scores indicate more severe anxiety and depressive symptoms.

### 2.2 MR image acquisition

A 3.0 T MRI scanner (Ingenia, Philips Medical Systems, Best, The Netherlands) equipped with an 8-channel head coil was used in this study. All participants underwent MRI scanning during the interictal period and were not taking preventive medications. Functional images were acquired in the axial plane using a gradient echo-planar imaging sequence. During resting-state fMRI acquisition, participants were instructed to remain still, keep their eyes closed, stay awake, and avoid engaging in any specific thoughts.

Resting-state functional images were acquired using the following parameters: repetition time (TR), 2000 ms; echo time (TE), 30 ms; flip angle (FA), 90°; 36 slices; slice thickness, 4 mm; no interslice gap; field of view (FOV), 240 × 240 mm; and acquisition matrix, 64 × 64. The total acquisition time for rs-fMRI was 8 min 8 s. As this study was based on a retrospective whole-brain resting-state fMRI dataset acquired for conventional whole-brain analysis rather than LC-optimized brainstem imaging, the spatial resolution was not specifically tailored for precise delineation of the locus coeruleus. High-resolution three-dimensional T1-weighted structural images were acquired using a turbo fast-echo sequence with the following parameters: TR, 8.1 ms; TE, 3.7 ms; 170 slices; slice thickness, 1 mm; no interslice gap; FA, 8°; acquisition matrix, 256 × 256; and FOV, 256 × 256 mm. Fluid-attenuated inversion recovery (FLAIR) images were also acquired to exclude structural abnormalities, using the following parameters: TR, 7000 ms; TE, 120 ms; 18 slices; slice thickness, 6 mm; interslice gap, 1.3 mm; FA, 110°; and voxel size, 0.65 × 0.95 × 6 mm^3^. After visual inspection of structural MRI scans, participants with brain tumors, cerebrovascular disease, hydrocephalus, or obvious white matter hyperintensities were excluded from further analysis.

### 2.3 MR image processing

The present study was based on a retrospective whole-brain resting-state fMRI dataset, and a conventional standardized preprocessing pipeline was applied to all participants to ensure methodological consistency and comparability in group-level analyses. Image preprocessing was performed using Statistical Parametric Mapping (SPM12; www.fil.ion.ucl.ac.uk/spm/software/spm12/) and the Conn toolbox (version 18b) [15], implemented in MATLAB R2013b (MathWorks, Natick, MA, USA). The preprocessing procedures included realignment and unwarping, slice-timing correction, segmentation into gray matter, white matter, and cerebrospinal fluid (CSF), normalization to the Montreal Neurological Institute (MNI) standard space, and spatial smoothing with an 8-mm full-width at half-maximum (FWHM) Gaussian kernel. To reduce the effects of head motion and other artifacts, ART-based scrubbing implemented in the Conn toolbox was used to identify outlier volumes. The motion threshold was set at 3 mm, and the global signal threshold was set at *Z* = 9. Nuisance variable regression was conducted, with the first five principal components from the segmented white matter and CSF removed from the signal. The six motion realignment parameters, their first-order derivatives, and the outlier volumes detected during the scrubbing procedure were similarly regressed out of the signal. The data then underwent linear detrending followed by bandpass filtering between 0.01 and 0.08 Hz. Although this conventional preprocessing pipeline is widely used in whole-brain rs-fMRI studies, it may be suboptimal for a structure as small as the LC and may therefore reduce anatomical specificity in this region.

Seed-to-voxel FC analysis was performed to investigate whole-brain FC patterns associated with the LC. Bilateral LC masks were defined according to Bär *et al*. [16]. Specifically, the left LC was defined as a 4 × 6 × 10 mm region centered at the MNI coordinates −5, −34, −21, and the right LC was defined as a 4 × 6 × 10 mm region centered at 7, −34, −21 (Fig. 1, Ref. [16]). These previously published atlas-based coordinates were used to promote anatomical consistency across participants in this retrospective dataset, in which subject-specific LC delineation was not available. Separate left and right LC masks were used as seed regions for subsequent FC analyses. Individual seed-to-voxel maps were generated for each participant using the LC seeds. Two-sample *t*-tests were performed within a default whole-brain mask to identify group differences in LC-related FC between patients with MwoA and HCs. A voxel-level statistical threshold of *p* < 0.01 was applied, followed by cluster-level correction using Gaussian random field theory with a two-tailed threshold of *p* < 0.05. Using RESTplus, significant positive clusters were transformed into binary masks, and mean FC strength values were subsequently extracted from these regions based on the z-maps. In light of the spatial resolution and preprocessing characteristics of the present dataset, these FC results were interpreted as reflecting LC-centered connectivity alterations at the network level rather than definitive abnormalities confined exclusively to the LC proper.

**Fig. 1.** The bilateral Locus Coeruleus seed masks. Masks were defined according to Bär (Bär *et al*. [16], 2016) (combined left and right LC; left LC seed was defined as 4 × 6 × 10 mm centered at MNI-coordinates −5, −34, −21, and right LC seed was defined as 4 × 6 × 10 mm centered at MNI-coordinates 7, −34, −21). Blue, left locus coeruleus; Red, right locus coeruleus.

### 2.4 Statistical analysis

Statistical analyses of demographic and clinical data were performed using SPSS version 17.0 (SPSS Inc., Chicago, IL, USA). Continuous variables were expressed as the mean ± standard deviation (SD), while categorical variables were expressed as counts. The normality of continuous variables was assessed using the Shapiro-Wilk test before statistical analysis. Between-group differences in demographic and clinical characteristics were assessed using independent-samples *t*-tests for continuous variables and chi-square tests for categorical variables. A value of *p* < 0.05 was considered statistically significant. Brain regions showing significant between-group differences were then selected for subsequent analyses of the associations between FC alterations and clinical variables in the MwoA group. Subsequently, average *z*-values were calculated for each participant within the aberrant FC region mask. To further explore the clinical significance of altered LC-related FC, Pearson correlation analyses were performed between these mean *z*-values and clinical variables only in the MwoA group, including migraine attack frequency, disease duration, SAS scores, and SDS scores, with adjustment for age, sex, and educational background. Finally, region-of-interest (ROI)-based group comparisons were performed using analysis of covariance (ANCOVA), followed by Bonferroni-corrected *post hoc* analyses where appropriate. These analyses were intended to examine within-patient dimensional associations rather than symptom-brain relationships in HCs; therefore, parallel correlation analyses were not performed in the HC group.

In addition, ROC curve analysis was performed in SPSS to evaluate the ability of FC values from brain regions showing significant between-group differences to discriminate patients with MwoA from HCs. For each ROC curve, sensitivity and specificity were calculated across all possible cutoff values of the corresponding FC measure, and the optimal cutoff value was determined according to the maximum Youden index. The area under the curve (AUC), sensitivity, and specificity were then reported accordingly. Sex, age, years of education, and mean gray matter volume were included as covariates in the group comparison analyses from which the altered FC values were derived. Due to the relatively limited sample size, no external validation set or cross-validation procedure was applied in the present study. Therefore, the ROC analysis should be considered exploratory, and the corresponding classification performance should be interpreted with caution.

## 3. Results

### 3.1 Demographic and clinical characteristics of participants

The demographic characteristics and clinical assessment results of all participants are summarized in Table 1. There were no significant differences between patients with MwoA and HCs in age, sex distribution, years of education, MoCA score, SAS score, or SDS score, as assessed using the chi-square test for sex and two-tailed independent-samples *t*-tests for the remaining variables (all *p* > 0.05). These findings indicate that the two groups were well matched in regard to the major demographic and baseline neuropsychological variables.

**Table 1.** The demographic and clinical characteristics of participants.

Characteristics	MwoA Patients (n = 55)	HCs (n = 50)	Mean difference (95% CI)	Effect size	*p*-value
Age (yr)	34.70 ± 9.77	36.83 ± 9.99	−2.13 (−5.96, 1.70)	−0.22	0.290
Education (yr)	13.97 ± 2.68	14.46 ± 2.85	−0.49 (−1.56, 0.58)	−0.18	0.461
Sex (male/female)	16:39	12:38	-	0.06^b^	0.924^a^
SAS	42.62 ± 10.59	39.24 ± 9.53	3.38 (−0.51, 7.27)	0.33	0.088
SDS	46.79 ± 12.01	43.37 ± 13.22	3.42 (−1.48, 8.32)	0.27	0.170
MoCA	26.07 ± 1.89	26.34 ± 1.69	−0.27 (−0.96, 0.42)	−0.15	0.616
HIT-6	57.60 ± 7.96	-	-	-	-
MIDAS	17.76 ± 7.37	-	-	-	-
Frequency (days per month)	4.07 ± 3.24	-	-	-	-
Duration (yr)	11.87 ± 8.29	-	-	-	-

*CI, Confidence Interval; HC, Healthy Control; HIT-6, Headache Impact Test-6; MIDAS, Migraine Disability Assessment; MoCA, Montreal Cognitive Assessment; MwoA, Migraine Without Aura; SAS, Self-rating Anxiety Scale; SDS, Self-rating Depression Scale. Values are represented as the mean ± SD. Unless otherwise indicated, p-values were calculated with 2-tailed t-tests. Effect sizes for continuous variables are reported as Cohen’s d. ^a^The p values were obtained using Chi-square test. ^b^The effect size for sex was calculated using the Phi coefficient.*

### 3.2 Resting-state functional connectivity maps of the LC seed regions

The resting-state FC maps of the bilateral LC seed regions in the MwoA and HC groups are shown in Fig. 2. In both groups, the left and right LC demonstrated distributed functional connectivity with widespread cortical and subcortical areas, including frontal, temporal, parietal, occipital, and cerebellar regions. This connectivity pattern was broadly consistent with the extensive anatomical and functional projections of the LC.

**Fig. 2.** Resting-state functional connectivity maps of the bilateral Locus Coeruleus. The Migraine patients, n = 55; the healthy controls, n = 50. L, left; R, right; LC, Locus Coeruleus.

### 3.3 Between-group differences in LC-centered FC

Compared with HCs, patients with MwoA showed abnormal LC-centered FC in several brain regions (Table 2 and Fig. 3). For the left LC seed, patients with MwoA exhibited significantly increased FC with the right superior temporal gyrus (STG) and left cerebellar posterior lobe (CPL), whereas significantly decreased FC was observed with the right superior frontal gyrus (SFG) (*p* < 0.05).

**Table 2.** Abnormal LC-centered FC in MwoA compared with HCs.

Brain region		Peak MNI coordinates			Voxel size	Peak *t* score
		X	Y	Z		
Left LC						
	R_STG	42	6	−21	40	4.5001
	R_SFG	27	42	39	145	−3.8026
	L_CPL	−24	−87	−39	60	3.5365
Right LC						
	R_SFG	27	42	42	201	−4.1711
	L_IOG	−30	−78	−6	98	3.6993
	L_STG	−42	15	−21	86	3.6002

*Significance was determined at a voxel-level threshold (p < 0.01) with Gaussian random field theory correction at a cluster-level threshold (two-tailed, p < 0.05). FC, Functional Connectivity; HC, Healthy Control; MwoA, Migraine Without Aura; LC, Locus Coeruleus; SFG, Superior Frontal Gyrus; IOG, Inferior Occipital Gyrus; STG, Superior Temporal Gyrus; CPL, Cerebellar Posterior Lobe; MNI, Montreal Neurological Institute.*

**Fig. 3.** Between-group differences in functional connectivity. Altered functional connectivity between the bilateral Locus Coeruleus and whole-brain regions in patients with migraine without aura (MwoA) compared with healthy controls. L, left; R, right; LC, Locus Coeruleus; SFG, Superior Frontal Gyrus; IOG, Inferior Occipital Gyrus; STG, Superior Temporal Gyrus; CPL, Cerebellar Posterior Lobe.

For the right LC seed, patients with MwoA showed significantly increased FC with the left inferior occipital gyrus (IOG) and left STG, while significantly decreased FC was again observed with the right SFG (*p* < 0.05). These findings indicate that MwoA is associated with altered LC-centered connectivity involving temporal, occipital, cerebellar, and prefrontal regions, with partly distinct but overlapping patterns between the left and right LC seeds.

### 3.4 Associations with the clinical characters

To explore the clinical relevance of LC-related FC abnormalities, mean *z*-values were extracted from all significant clusters showing altered FC with the LC (Table 2; Figs. 2,3). Pearson correlation analyses were then performed within the MwoA group between these extracted FC values and clinical measures, including migraine attack frequency, disease duration, SAS scores, and SDS scores.

Most altered FC measures were not significantly correlated with the examined clinical variables. However, two significant positive correlations were identified. FC between the right LC and left IOG was positively correlated with SDS score (*p* = 0.011, *r* = 0.344), suggesting a potential association between LC-occipital connectivity and depressive symptom burden in patients with MwoA. In addition, FC between the left LC and right STG was positively correlated with disease duration (*p* = 0.012, *r* = 0.341) (Fig. 4).

**Fig. 4.** Correlations between the functional connectivity of the Locus Coeruleus and the clinical characteristics. (A) Positive correlation between SDS score and FC of the right LC–left IOG connection (*p* = 0.011, *r* = 0.344). (B) Positive correlation between disease duration and FC of the left LC–right STG connection (*p* = 0.012, *r* = 0.341). L, left; R, right; LC, Locus Coeruleus; SFG, Superior Frontal Gyrus; IOG, Inferior Occipital Gyrus; STG, Superior Temporal Gyrus; CPL, Cerebellar Posterior Lobe; SDS, Self-Rating Depression Scale; FC, functional connectivity.

### 3.5 ROC analysis for differential diagnosis

ROC analyses were performed using FC values extracted from the abnormal clusters to evaluate their ability to distinguish patients with MwoA from HCs. The AUC values for all examined clusters were ≥0.696, and the corresponding AUC, sensitivity, and specificity values are presented in Fig. 5. Among these altered connectivity measures, FC between the right LC and right SFG showed the strongest discriminatory performance, with a sensitivity of 81.82%, a specificity of 68.00%, and an optimal resting-state FC cutoff value of −0.012.

**Fig. 5.** ROC analysis for differential diagnosis. The ROC curves of significant ROI of LC FC in the MwoA group and the HC group. ROC, receiver operating characteristic; ROI, region of interest; LC, Locus Coeruleus; FC, functional connectivity; MwoA, Migraine without aura; HC, healthy control; L, left; R, right; STG, Superior Temporal Gyrus; CPL, Cerebellar Posterior Lobe; SFG, Superior Frontal Gyrus; IOG, Inferior Occipital Gyrus.

## 4. Discussion

It is well established that migraine involves activation and sensitization of the trigeminovascular pathway, along with involvement of brainstem and diencephalic nuclei [17]. The LC, a nucleus located in the dorsolateral brainstem, plays a pivotal role in regulating sympathetic nervous system activity [18].

In the present study, patients with MwoA showed altered LC-centered resting-state FC with regions implicated in pain processing, emotional regulation, and sensory integration. Within this interpretive framework, we further observed positive associations between altered LC-related connectivity and disease duration as well as depressive symptom severity, together with moderate discriminatory performance of right LC–right SFG connectivity in the exploratory ROC analysis. Overall, these findings provide additional evidence for LC-centered network abnormalities in MwoA. Our findings extend the existing literature [8, 9, 10] by providing a more refined whole-brain characterization of bilateral LC-centered FC abnormalities in a specifically defined MwoA cohort, with separate analyses of the left and right LC and additional exploration of their clinical and preliminary discriminatory relevance.

A principal finding was altered FC between the LC and the cerebellum, as well as the SFG. Beyond its classical role in motor coordination, the cerebellum has also been implicated in nociceptive processing and multisensory integration [19]. Studies have shown that cerebellar dysfunction can impair pain suppression mechanisms, thereby exacerbating nociceptive hypersensitivity in patients with migraine [20, 21, 22]. The observed increased FC between the left LC and left CPL may reflect altered functional coupling within pain- and sensory-related networks in MwoA. We also found decreased FC between the bilateral LC and the right SFG. The superior frontal gyrus, a key component of the prefrontal cortex, modulates pain through top-down mechanisms and is also related to cognitive and emotional functions [23, 24]. This finding may indicate altered functional coupling between brainstem noradrenergic regions and prefrontal cortical areas. In addition, increased FC was observed between the LC and the STG, as well as between the right LC and the left IOG. STG and IOG are involved in auditory and visual processing, respectively [25, 26]. Considering that migraine is commonly accompanied by phonophobia and photophobia, these altered connectivity patterns may be related to abnormal sensory integration in migraine. Together, these results suggest that LC-centered alterations in migraine may extend beyond pain-related circuits to broader sensory networks. However, considering the spatial resolution and preprocessing characteristics of the present data, these findings are more appropriately interpreted as LC-centered or peri-LC network alterations rather than precise evidence of isolated LC nucleus dysfunction.

Regarding clinical relevance, another notable finding was the positive correlation between disease duration and FC between the left LC and the right STG. This suggests that LC-related functional alterations may be associated with the long-term clinical course of MwoA. Previous longitudinal studies have reported structural and functional reorganization in pain-related networks among patients with chronic pain, including those with migraine [27, 28]. Our findings may therefore reflect cumulative network-level changes associated with repeated migraine episodes.

In addition, although a positive correlation between SDS score and FC between the right LC and the left IOG was observed within the MwoA group, this result should be interpreted cautiously. As SDS scores did not significantly differ between patients with MwoA and HCs, the FC-SDS association may be better interpreted as a within-patient dimensional association in MwoA rather than a disease-specific alteration. Nevertheless, given that migraine is increasingly recognized as a heterogeneous brain disorder with manifestations extending beyond pain alone [29], this result may still be clinically informative by indicating that LC-related FC is associated with variability in emotional symptoms across patients [30, 31]. Although preliminary, this observation underscores the importance of considering affective dimensions in a more comprehensive evaluation of MwoA, and its potential relevance for phenotypic characterization or individualized assessment warrants further investigation in larger and longitudinal cohorts.

ROC analysis showed that altered FC patterns had some ability to distinguish patients with MwoA from HCs. Among these patterns, FC between the right LC and the right SFG showed the best discriminatory performance, with a sensitivity of 81.82% and a specificity of 68.00%. However, as the corresponding AUC indicated only moderate discrimination, these results suggest that LC-related FC alterations may have preliminary imaging relevance for distinguishing patients with MwoA from healthy individuals.

## 5. Limitations

This study has several important limitations. First, the modest sample size and cross-sectional design limit the generalizability of the findings and prevent conclusions regarding the longitudinal evolution of LC-related FC changes in MwoA. Second, methodological constraints related to LC imaging should be considered. Because the LC is a small brainstem nucleus, the conventional spatial resolution and preprocessing pipeline used in this retrospective whole-brain rs-fMRI dataset may have reduced localization precision and introduced partial-volume effects or signal contamination from adjacent pontine structures. Accordingly, the observed abnormalities are better interpreted as LC-centered network alterations rather than definitive nucleus-specific LC findings. Third, the clinical association analyses were exploratory. These analyses were performed only within the MwoA group and used FC clusters defined by between-group differences; moreover, the correlation between SDS score and right LC–left IOG connectivity should be interpreted with caution because SDS scores did not differ significantly between patients and controls. Fourth, the ROC results should also be viewed as preliminary, as no external validation set or cross-validation procedure was applied. Future studies should recruit larger samples, adopt longitudinal designs, and incorporate multimodal or LC-optimized imaging approaches, including higher spatial resolution, reduced or no smoothing in brainstem-focused analyses, and subject-specific LC localization, to further validate and refine the present findings.

## 6. Conclusions

In conclusion, this study demonstrated altered LC-centered resting-state FC in patients with MwoA, involving brain regions associated with pain processing, sensory integration, and emotional regulation. By separately examining the left and right LC, the present study extends previous LC-related imaging findings in migraine and provides a more refined characterization of bilateral LC-centered network dysfunction in a specifically defined MwoA cohort. The exploratory ROC results further suggest that altered LC-related FC may have preliminary imaging relevance for distinguishing patients with MwoA from HCs, although these findings require validation in larger independent cohorts before any diagnostic application can be considered. Overall, our results provide additional evidence that LC-related network alterations may contribute to the neurobiological features of MwoA and may help inform future longitudinal, multimodal, and mechanistic studies.
